# Thai and American mothers socialize preschoolers’ emotional development differently

**DOI:** 10.1038/s41598-023-39947-0

**Published:** 2023-08-05

**Authors:** Sirada Rochanavibhata, Viorica Marian

**Affiliations:** Department of Communication Sciences and Disorders, Northwestern University, 2240 Campus Drive, Evanston, IL 60208-3540 USA

**Keywords:** Human behaviour, Psychology

## Abstract

Cultures vary in beliefs about appropriate display of emotion. Children rely on adults to help them understand emotional experiences and display emotions in a culturally appropriate manner. The present study compared how emotion display differs between Thai and American mother–child interactions during preschool. Language samples from 21 Thai and 21 American mother–child dyads were elicited using prompted reminiscing, book reading, toy play, and child personal narrative tasks. Results revealed group differences in emotion talk and behavior. American dyads expressed more intense emotions during interactions compared to Thai dyads. American dyads also displayed more emotion behaviors than Thai dyads, whereas Thai dyads used more emotion words compared to American dyads. Additionally, there were gender differences in the expression of emotion, with boy dyads more emotionally intense than girl dyads in both groups. Boys displayed more negative emotion behaviors compared to girls during prompted reminiscing, whereas girls used more negative emotion words than boys during the personal narrative task. These findings demonstrate cultural and gender differences in socialization goals and practices regarding emotion display and underscore the influence of mothers’ scaffolding on children’s emotional development. This research reveals the variability in beliefs and values that underlie emotional development across sociocultural contexts.

## Introduction

Children learn culturally appropriate ways of interacting with their social world from their caregivers^1–5^, including ways of displaying and talking about emotions^6^. One of the common contexts in which adults model emotion behaviors and transfer emotion knowledge is adult-child conversation. The ways that caregivers socialize children differ depending on culture-^7^ and gender-specific norms^8,9^ for emotion display. Emotion socialization during preschool is especially crucial in influencing child emotional and social competence^10–12^. To date, our understanding of emotional development is drawn from research focused primarily on WEIRD Western, educated, industrialized, rich, democratic populations. The present study examined the influence of cultural background and child gender on Thai and American maternal scaffolding of their preschoolers’ emotional development. This work is the first to examine emotion socialization of Thai preschoolers across four naturalistic parent-child communicative settings. By focusing on an underrepresented population in developmental science, findings from the present research contribute to our understanding of the influence of culture on emotion socialization.

### Emotion socialization throughout development

Although genetics influence children’s temperament, studies have shown that environmental factors, including interactions with caregivers, play important roles in shaping children’s expressions of positive and negative affect^13–15^. Parental scaffolding of emotion, also referred to as emotion socialization, is comprised of three primary processes: caregivers’ discussion of emotion with children, caregivers’ reaction to children’s emotion, and caregivers’ own expression of emotion^6^. Accordingly, parent-child conversations about emotions have been shown to predict children’s understanding of emotions^16–18^. For instance, mothers’ emotion labels during storytelling, as well as questions about and references to children’s emotions during dyadic reminiscing, are associated with better emotion understanding in preschoolers^19^. Additionally, children learn about emotions via seeing examples of others’ emotional expressions and responses to various emotional displays^10^. Parents who express positive affect have children who also express more positive emotions^20–22^. On the other hand, parents who express negative affect, including anger and tenseness, have children who are high in negative emotionality^23,24^. Thus, children’s emotional development is socialized verbally by adult emotion talk and non-verbally by adult emotion display.

Although the development of emotional competence occurs from early childhood through adolescence, acquiring socioemotional skills during the preschool years is particularly important. During this period, children start to understand emotions and use emotion language^11^. They also start to interact regularly with others outside the home and must learn to regulate their emotions and maintain appropriate emotional displays^10–12^. Therefore, researchers have commonly studied emotion socialization by parents of preschoolers^25–29^.

### Cultural differences in emotion socialization

As with other socialization processes, the socialization of children’s emotional development is culturally bound^7,30–32^. Children are taught to express and understand emotions in ways that are congruent with socially valued behaviors and outcomes. During dyadic interactions, the emotion words used by adults transfer culturally relevant knowledge about emotion concepts^33–36^. Eastern and Western cultures differ on values associated with collectivism and individualism. Because Asian cultures tend to emphasize compliance with social norms and maintenance of group cohesion, emotional expression is typically less encouraged^37^. Conversely, European American culture values individuality and self-expression, which means that expressions of thoughts and feelings tend to be encouraged^38,39^. Cross-cultural comparisons of mother-child interactions have indeed provided evidence in support of these differences. For instance, Chinese mothers tend to discuss emotions less often than European American mothers^40,41^. When emotions are discussed, Chinese mothers do so while teaching their children the social rules related to emotional displays^29,40,41^.

Emotion behaviors, specifically behavioral displays that convey positive or negative emotions, also differ across cultures. Mothers model appropriate emotion behaviors during interactions with their children. For example, European American mothers tend to display more positive facial expressions compared to Chinese mothers^7^. There is also evidence showing that children display emotions differently as a function of their cultural background. European American children display happiness, sadness, and anger more frequently than Korean and Asian American children^42,43^. Cultural expectations are also reflected in social reactions to children’s emotion behaviors. Among the same group of children, Korean and Asian American children’s expressions of happiness and sadness are associated with negative ratings of social competence by adults, which suggests that children from Eastern backgrounds are expected to be less expressive when showing happiness and sadness.

Lastly, cultures vary in their preferred emotional valence and intensity. Specifically, European American and Asian cultures have been shown to differ on these two dimensions of emotion. Relative to Indian American and Chinese mothers, European American mothers express more positive affect^44,45^ and less negative or neutral affect^44^. When expressing the same type of affect, the ideal intensity of that affect can also differ across cultures. European Americans tend to prefer high intensity affect while Asians tend to prefer low intensity affect^46,47^.

### Gender differences in emotion socialization

In addition to culture-based norms and expectations, socialization goals vary as a function of child gender. Parents’ references to emotion are more frequent and varied with daughters than with sons^34,48^. Specifically, they discuss sadness, dislike, and fear more often with daughters than with sons^34,49,50^. On the other hand, mothers tend to discuss being angry with their sons^50^. As a result of adult socialization, girls talk more about their emotions and use more emotion words when discussing scary experiences^34^. However, much of the previous research has examined the role of gender on emotion socialization in parent-child dyads from WEIRD populations. Considering that there are cross-cultural differences in values and beliefs regarding emotion display, it is possible that gender-specific socialization goals found in WEIRD populations may not be generalizable to understudied non-WEIRD cultures.

Among the studies that focus on emotion scaffolding in culturally diverse groups, results reveal inconsistent patterns of gender-specific socialization goals. For example, similar to European American mothers, Chinese mothers discuss emotions more frequently with daughters than with sons^32,41^. Consequently, Chinese girls recount emotional experiences more than boys^32^. These findings provide evidence that Eastern and Western cultures may share similar gendered socialization goals, specifically for girls to be emotionally expressive. However, other studies have found that Chinese and Turkish mothers encourage and focus on emotional expressions from boys more than from girls^51,52^. These incongruent patterns of gender-specific socialization goals suggest that perhaps cultural norms play a role in moderating how girls and boys are taught to express and understand emotion. However, to date, research that examined the interaction between culture and gender on emotion socialization has not found a significant moderating effect of culture^40,53^. Due to the limited number of studies examining emotional development in non-WEIRD populations and the lack of diverse cultural representation in the developmental science literature, the existing theories and knowledge of emotion socialization are incomplete. More research is warranted to better understand the roles that culture- and gender-specific norms play in influencing children’s emotional development, particularly among underrepresented groups.

### Emotion socialization in Thai culture

Cross-cultural differences in emotion socialization have primarily been examined in collectivist Eastern and individualistic Western cultures (e.g., Chinese vs. European American). Despite the shared values and norms, not all collectivist cultures are alike. It is not always possible to generalize findings from one culture to another. No studies to date have examined emotion socialization among Thai parent-preschooler dyads. Thai culture presents a unique opportunity for cross-cultural comparison. On Hofstede’s individualism–collectivism continuum^54^, Thailand has an index score of 17 (indicative of a highly collectivist culture), whereas the United States has an index score of 91 (indicative of a highly individualistic culture). Additionally, cross-cultural studies have consistently shown that Thais define their self-construal in relation to others (i.e., interdependent self-construal)^55,56^. Like other collectivist and interdependent Asian countries, Thai culture places an emphasis on good conduct, social relationships, and group harmony. Moreover, the pervasiveness of collectivist values in Thailand is also reflected in its official language^57^. Thai has honorific terms that are used to convey social status and to show respect (e.g., terms that specify whether one’s interlocutor is younger or older). Honorific particles—words that are added to the end of an utterance—are also used to show politeness to the person being addressed. However, there are core values and teachings that set Thailand apart from its other collectivist counterparts. Children are taught from early childhood the concept of “kreng jai,” meaning “to have consideration for,” which instills a mindset of self-effacement and humility that aims to minimize disturbance to others^58^. There is also the common phrase “jai yen,” which can be translated to “cool heart” and describes the common Thai quality of remaining calm and in control of one’s emotions^59^. As a result of these values, public displays of extreme emotion are considered taboo in Thai society^60^.

The extant literature on Thai cultural practices has primarily examined emotion regulation and display rules. For example, Thai parents tend to be intolerant of children’s “undercontrolled” behavior including aggressive, disobedient, and disrespectful acts^58,61^. Thai children are also taught to inhibit outward expression of anger and other strong emotions^62^. To our knowledge, no studies have focused on the ways through which Thai adults socialize children on expression of positive and negative valence emotions or discrete emotions (e.g., happiness, sadness, fearfulness, anger). Considering the lack of previous research on the primary processes of emotion socialization (i.e., caregivers’ discussion of emotion with children, caregivers’ reaction to children’s emotion, and caregivers’ own expression of emotion^6^) in Thai culture, the first step towards a better understanding of emotion socialization in an understudied population is to observe, record, and document naturalistic parent-child interactions.

### The present study

Because Thai children are often taught to be polite and obedient, to show self-control, and to refrain from displaying strong emotions^58–62^, there is reason to believe that Thai and American children may be socialized to discuss and display emotion differently. The present study focused on emotion socialization in Thai and American mother-child dyads by comparing the emotion intensity, emotion words, and emotion behaviors during four tasks—reminiscing, book sharing, toy play, and child personal narrative. The same datasets as reported in previous work on Thai and American mother-child dyads^63–65^ were examined; emotion data presented here have not been previously reported.

Congruent with the cultural and societal norms associated with the individualistic American culture and collectivist Thai culture, we expected American mother-child dyads to have interactions that were higher in emotional intensity compared to Thai mother-child dyads. During the personal narrative task, we also expected American children to have interactions that were higher in emotional intensity compared to Thai children, even without their mothers’ presence. We expected for American mothers and children to use more emotion words (both positive and negative) and display more emotion behaviors (both positive and negative) than Thai mothers and children. When comparing across genders, we expected girl dyads to have interactions that were higher in emotional intensity compared to boy dyads, and for girls’ interactions with the experimenter to be more emotionally intense than boys’. We also predicted girls and their mothers to use more emotion words and display more emotion behaviors than boys and their mothers.

## Results

Maternal and child data were analyzed with 2 (culture) × 2 (child gender) ANOVAs. Means, standard deviations, and statistical significance are presented in Tables 1, 2, 3, 4. See Table 5 for a summary of significant cross-cultural differences in emotion display across the four tasks.

**Table 1:** Mean percentages (standard deviations) of maternal and child emotion measures across cultures and child gender during prompted reminiscing.

Emotion measure	Culture		*F* value	Child gender		*F* value	Interaction *F* value
	Thai	American		Boy	Girl		
Dyad							
Intensity	3.73 (0.73)	4.21 (0.88)	3.57^†^	4.24 (0.85)	3.70 (0.75)	4.64*	0.37
Mothers							
Words							
Positive	1.13 (0.84)	0.73 (0.32)	3.77†	0.89 (0.72)	0.97 (0.61)	0.14	0.20
Negative	0.37 (0.15)	0.22 (0.15)	4.68*	0.30 (0.25)	0.29 (0.23)	0.14	0.20
Behaviors							
Positive	0.43 (0.35)	0.63 (0.36)	3.69†	0.41 (0.25)	0.64 (0.43)	5.35*	0.06
Negative	0.001 (0.01)	0.01 (0.03)	1.71	0.01 (0.02)	0.01 (0.02)	0.45	0.01
Children							
Words							
Positive	1.06 (0.70)	0.57 (0.47)	4.03^†^	0.72 (0.71)	0.91 (0.57)	0.86	1.16
Negative	0.66 (0.50)	0.36 (0.30)	5.52*	0.48 (0.43)	0.54 (0.44)	0.19	0.59
Behaviors							
Positive	0.44 (0.34)	1.46 (1.41)	8.14**	0.86 (1.32)	1.03 (0.94)	0.09	0.15
Negative	0.19 (0.29)	0.26 (0.41)	0.39	0.35 (0.41)	0.10 (0.23)	5.14*	0.01

^†^*p* < .10, *p < .05, **p < .01.

**Table 2:** Mean percentages (standard deviations) of maternal and child emotion measures across cultures and child gender during book sharing.

Emotion measure	Culture		*F* value	Child gender		*F* value	Interaction *F* value
	Thai	American		Boy	Girl		
Dyad							
Intensity	3.76 (0.77)	4.38 (1.07)	4.36*	4.29 (0.90)	3.86 (1.01)	1.94	0.23
Mothers							
Words							
Positive	0.25 (0.26)	0.38 (0.31)	1.78	0.40 (0.32)	0.23 (0.25)	3.63^†^	1.86
Negative	0.93 (0.59)	0.74 (0.45)	0.91	0.75 (0.45)	0.93 (0.59)	1.28	0.01
Behaviors							
Positive	0.23 (0.18)	0.52 (0.50)	5.71*	0.41 (0.42)	0.33 (0.38)	0.28	0.32
Negative	0 (0)	0.07 (0.13)	5.19*	0.04 (0.12)	0.03 (0.08)	0.43	0.43
Children							
Words							
Positive	0.25 (0.46)	0.29 (0.43)	0.01	0.30 (0.44)	0.25 (0.44)	0.04	0.53
Negative	0.84 (1.51)	1.23 (1.05)	0.48	0.96 (1.07)	1.11 (1.52)	0.29	1.86
Behaviors							
Positive	2.36 (5.25)	2.00 (2.55)	0.31	2.38 (5.26)	1.98 (2.52)	0.57	0.36
Negative	0.16 (0.50)	0.03 (0.11)	1.07	0.11 (0.37)	0.08 (0.36)	0.44	0.002

^†^*p* < .10, *p < .05.

**Table 3:** Mean percentages (standard deviations) of maternal and child emotion measures across cultures and child gender during toy play.

Emotion measure	Culture		*F* value	Child gender		*F* value	Interaction *F* value
	Thai	American		Boy	Girl		
Dyad							
Intensity	4.00 (1.18)	4.52 (1.21)	1.88	4.48 (1.36)	4.05 (1.02)	1.21	1.21
Mothers							
Words							
Positive	0.20 (0.17)	0.24 (0.19)	0.35	0.20 (0.18)	0.24 (0.18)	1.03	0.01
Negative	0.34 (0.43)	0.36 (0.32)	0.03	0.34 (0.38)	0.36 (0.38)	0.01	1.22
Behaviors							
Positive	0.72 (0.50)	1.04 (0.80)	2.62	0.78 (0.54)	0.98 (0.79)	1.32	0.05
Negative	0 (0)	0.02 (0.07)	1.89	0.01 (0.04)	0.01 (0.06)	0.35	0.35
Children							
Words							
Positive	0.32 (0.51)	0.16 (0.20)	1.06	0.22 (0.32)	0.26 (0.46)	0.47	0.09
Negative	0.40 (0.53)	0.17 (0.21)	2.54	0.31 (0.39)	0.27 (0.44)	0.003	0.03
Behaviors							
Positive	1.03 (1.35)	1.26 (1.23)	0.15	1.47 (1.51)	0.82 (0.93)	2.33	0.10
Negative	0.03 (0.08)	0.15 (0.18)	5.83*	0.08 (0.12)	0.11 (0.18)	0.33	1.13

*p < .05.

**Table 4:** Mean percentages (standard deviations) of child emotion measures across cultures and child gender during personal narrative.

Emotion measure	Culture		*F* value	Child gender		*F* value	Interaction *F* value
	Thai	American		Boy	Girl		
Children							
Intensity	2.76 (0.97)	3.73 (1.43)	7.10*	3.67 (1.27)	2.81 (1.20)	5.54*	3.09^†^
Words							
Positive	0.01 (0.03)	0.07 (0.17)	1.93	0.05 (0.13)	0.03 (0.12)	0.006	0.11
Negative	3.04 (2.69)	1.39 (1.43)	6.66*	1.51 (1.88)	2.91 (2.49)	6.88*	0.97
Behaviors							
Positive	0.14 (0.45)	0.08 (0.21)	0.39	0.06 (0.19)	0.16 (0.45)	0.99	2.91
Negative	0.12 (0.53)	0.04 (0.13)	0.56	0.14 (0.53)	0.02 (0.09)	0.98	0.78

^†^*p* < .10, *p < .05.

**Table 5:** Summary of significant cross-cultural differences in emotion display across tasks.

Emotion measure	Prompted reminiscing	Book sharing	Toy play	Personal narrative
Dyad				
Intensity	–	American > Thai	–	American > Thai
Mothers				
Words				
Positive	–	–	–	N/A
Negative	Thai > American	–	–	N/A
Behaviors				
Positive	–	American > Thai	–	N/A
Negative	–	American > Thai	–	N/A
Children				
Words				
Positive	–	–	–	–
Negative	Thai > American	–	–	Thai > American
Behaviors				
Positive	American > Thai	–	–	–
Negative	–	–	American > Thai	–

### Emotional intensity

American mother-child dyads’ interactions were more emotionally intense compared to Thai dyads’ during the book reading task (η^2^ = 0.10). During their interaction with the experimenter, American children were also more emotionally intense than Thai children (η^2^ = 0.16). Boy dyads produced narratives that were more emotionally intense than girl dyads during the prompted reminiscing task (η^2^ = 0.11). Likewise, boys’ interactions with the experimenter during the personal narrative task were more emotionally intense than girls’ interactions (η^2^ = 0.13). There were no significant interactions between culture and gender on emotional intensity in any of the tasks.

### Use of emotion words

Thai mothers used negative emotion words significantly more (η^2^ = 0.11) and positive emotion words marginally more than American mothers during prompted reminiscing. Compared to American children, Thai children used negative emotion words more during prompted reminiscing (η^2^ = 0.13) and personal narrative tasks (η^2^ = 0.15) and used positive emotion words marginally more during the prompted reminiscing task.

Girls used more negative emotion words than boys during the personal narrative task (η^2^ = 0.15). Mothers of boys used positive emotion words marginally more than mothers of girls during book reading. There were no significant interactions between culture and gender on maternal and child use of positive and negative emotion words in any of the four tasks.

### Display of emotion behaviors

American mothers displayed positive emotion behaviors significantly more than Thai mothers during book sharing (η^2^ = 0.13) and marginally more than Thai mothers during prompted reminiscing. American mothers also displayed negative emotion behaviors significantly more than Thai mothers during the book sharing task (η^2^ = 0.12). American children displayed positive emotion behaviors more than Thai children during prompted reminiscing (η^2^ = 0.18) and displayed negative emotion behaviors more than Thai children during toy play (η^2^ = 0.13).

Mothers of girls displayed positive emotion behaviors more than mothers of boys (η^2^ = 0.12) and boys displayed negative emotion behaviors more compared to girls during prompted reminiscing (η^2^ = 0.12). There were no significant interactions between culture and gender on maternal and child display of positive and negative emotion behaviors in any of the tasks.

## Discussion

The present study examined the influence of culture and gender on emotion socialization within naturalistic settings in the home. Results revealed that Thai and American mothers modeled and scaffolded emotion talk and behavior differently. Children from the two cultural groups also differed in the ways they discussed and displayed emotions. Additionally, findings provided evidence for gendered socialization goals related to how children were expected to engage with emotions. This work provides insight into the socioemotional development of children from understudied populations during the critical preschool years.

The findings that American dyads’ interactions were more emotionally intense than Thai dyads’ interactions and that American mothers and children displayed both positive and negative emotions more than their Thai counterparts suggest that the cultural values related to childrearing practices influence emotion socialization. Self-expression is valued in American culture^38,39^, whereas self-control and restraint are valued qualities in Thai culture^58,62^. These results also suggest that similar to other Asian cultures, such as Chinese, Japanese, and Korean^46,47^, Thais prefer lower intensity affect more than European Americans. It is noteworthy that global emotional intensity judgments in this study were made based on the emotionality in the *voice* and emotional *content* of conversation (the extent to which narratives revolved around emotions). Emotion cues from facial expressions were not included in the judgment of emotional intensity as they were accounted for by the emotion behaviors measure. Future studies would benefit from using a more nuanced coding scheme, in which the emotional intensity judgment is made based on the number and quality of emotion cues extracted from several channels including the voice, face, and body posture. Considering both the auditory and visual modalities would improve the validity of the emotional intensity measure because emotions are often expressed and perceived multi-modally. Although facial and postural cues were not included in the emotional intensity ratings, the observed differences between groups in vocal emotionality and content of conversation have implications for practitioners working with culturally diverse children. In comparison to European American children, immigrant children from Asian cultures may be viewed as shy, quiet, or introverted if evaluated through the mainstream European American normative lens. It is important for clinicians and educators to understand culture-specific norms pertaining to emotion display in order to avoid misdiagnoses of children from diverse groups.

Thai mothers and children produced negative emotion words more than American mothers and children, which is incongruent with previous findings that Asian mothers tend to discuss emotions less often than European American mothers^40,41^. This pattern of emotion talk suggests that not all Asian cultures are alike. Previous research has found that even among groups that are influenced by the same traditional Chinese values (e.g., Taiwanese families in Taiwan, Chinese families in China, and Chinese American families in the United States), emotion is socialized differently^32,66^. For example, Taiwanese children use negative emotion words more than Chinese American children^66^. Considering that we did not directly compare Thai and Chinese dyads, the presents findings cannot speak to the differences in emotion socialization between the two Asian cultures. However, findings from the present study suggest that there are nuanced differences between cultures that fall on the same end of the individualism–collectivism continuum^67^. Understanding emotion socialization in understudied populations is necessary for developing more accurate and complete theories of human development.

Emotion socialization and display also differed as a function of child gender. We found that boy dyads produced narratives that were more emotionally intense than girl dyads. Previous research in adults has shown contrary evidence where women report experiencing more intense emotions than men^46^. The incongruence between findings from the present study and previous work could potentially be attributed to the fact that in our study, emotional intensity was evaluated by external raters for both boy and girl dyads, whereas in past research, men and women rated their own emotions. When reporting one’s own emotions, societal expectations regarding gender may influence how women and men respond. Specifically, given that women are socialized to be more emotionally expressive than men, it is possible that women subjectively report higher emotional intensity and men report lower emotional intensity to conform to those expectations. In contrast, objective raters who are blind to the hypotheses of the present study may not be subject to the same societal pressures. To our knowledge, the present study is the first to examine gender differences in emotional *intensity* among parent-child dyads, particularly among Thai and American dyads. Future work examining emotional intensity in naturalistic parent-child interactions is necessary to better understand how children across diverse cultural contexts are socialized based on their gender.

The girls in our study used more negative emotion words than boys, a finding consistent with previous research that demonstrates a tendency for European American girls to reference their emotions more than boys, particularly negative emotions such as sadness, dislike, and fear^34,49,50^. However, boys displayed more negative emotion behaviors than girls. The patterns for use of emotion words and displays of emotion behaviors provide evidence that adults may have different gendered socialization goals in mind for each type of emotional expression. It may be more acceptable or even expected for boys to *display* negative emotion behaviors^51,52^ and for girls to *talk* about their emotions^34,49,50^. Across the emotion measures and tasks, there were no significant interactions between culture and gender. These findings suggest that culture may not moderate gender differences in emotion to a great extent^40,53^ and that gender-based socialization goals regarding emotion may be relatively uniform across cultures.

Notably, compared to the other tasks, toy play was the task with the most homogeneity across the two cultural groups in terms of emotion talk and behavior. Specifically, the only cross-cultural difference observed was that American children displayed more negative emotion behaviors than Thai children. The fact that emotion intensity, talk, and behavior—particularly mothers’ use of emotion words and behaviors—were similar across the two groups suggest that toy play may not be as conducive to scaffolding and modeling emotion display compared to the reminiscing and book sharing tasks. However, it is important to note that the farm animal toy pieces used in this study were culture- and gender-neutral. Thus, the generalizability of the current findings to other types of toy play is to be determined. It is possible that a toy play task involving stereotypically gendered toys may reveal more heterogeneity in emotion socialization.

Nevertheless, the homogeneity across cultures observed during toy play in this study may be the reason why previous researchers have primarily examined cross-cultural differences in emotion during naturalistic settings that are more narrative centered (e.g., reminiscing or book sharing^40,41,53^). In the reminiscing task, mothers and children recounted a variety of personal experiences, which naturally led to discussions of their own emotions. In the book sharing task, the storylines in the wordless picture books provided opportunities for mothers and children to discuss the emotions of the characters. Consequently, in these tasks, cultural differences in emotion intensity, talk, and behavior were observed. However, the toy play task consisted of farm animals, which might not have provided as much emotion content for dyads to discuss. Additionally, both narrative tasks were relatively more structured than the toy play task. For instance, mothers and children needed to sit and jointly attend to the book, while they were free to move around when playing with the toy pieces. As a result, the unstructured nature of the toy play task might have been conducive to eliciting emotion behaviors, but not emotion talk. Thus, researchers studying emotion socialization need to consider the aspects of emotion (e.g., use of emotion words or display of emotion behaviors) that they would like to examine when selecting the communicative settings in which to observe parent-child interactions.

One important caveat to note is that the interpretations and conclusions about American and Thai mothers’ socialization goals and cultural family models (i.e., individualistic versus collectivist) in the current work were made based on the observed mother-child dyadic interactions. This approach is consistent with the emotion socialization model by Eisenberg et al.^6^, which considers parents’ own emotional expression and discussion of emotion in the presence of the child as emotion socializing behaviors. Notably, these socializing behaviors may be produced with or without parents’ explicit intention to influence their children’s behaviors^68^. Additionally, in accordance with previous cross-cultural comparisons of parent-child emotion conversations^29,32^, the measures of maternal identification with individualistic/collectivist beliefs and values relied on self-reported cultural identification (e.g., American and Chinese). In the present research, families were informed that the objective of the study was to examine how children communicate with their mothers. We purposefully did not reveal that the objective of the research was to see how mothers socialize their children because we wanted to ensure that the dyadic interactions were as naturalistic as possible. We also did not want the mothers to be cognizant of cultural family models, specifically where they fell on the individualism–collectivism continuum, so as to not change their behaviors. As their demographic and linguistic profiles suggest, American and Thai mothers in the study reported high identification with their respective cultures. Because American and Thai cultures have been associated with individualism and collectivism, respectively^54^, the results were interpreted using the individualism–collectivism framework. A potential direction for future research could be to collect questionnaire data on parental beliefs regarding emotion socialization goals and cultural family models. Asking participants about their identification with individualistic and collectivist ideologies would allow researchers to determine whether implicit beliefs are congruent with explicit behavioral patterns.

Because children’s social sphere consists of various people both inside and outside the home, future research will need to examine emotion socialization by individuals other than children’s mothers, such as fathers, older siblings, grandparents, peers, and childcare providers^7^. It is also important to note the reciprocal nature of emotional exchanges between a parent and a child. Children’s emotionality affects parental socialization of emotion behaviors. For example, mothers’ positive emotion has been found to increase over time for children who were more content and less angry^69^. Considering the cross-sectional nature of this study, it is not possible to determine the extent to which maternal emotional scaffolding can be attributed to each child’s own temperament as opposed to the mothers’ own socialization goals. We are also unable to draw conclusions on the degree to which children’s expressions of emotion are accounted for by nature versus nurture. Longitudinal work that examines the trajectory of emotion socialization over time and carefully controls for the influence of genetics is necessary to draw definite causal relationships between maternal and child emotion display.

In sum, the present study demonstrated the influence of culture and gender on preschool children’s socioemotional development, particularly in the intensity of their emotions, use of positive and negative emotion words, and display of positive and negative emotion behaviors. By engaging in discussions about emotion, responding to children’s emotions, and expressing their own emotions, adults teach children how to conduct themselves appropriately in their social environment. Such a socialization process is particularly important during the preschool years when children undergo developmental changes with respect to their emotion understanding, as well as changes in their daily routine that require the ability to regulate their emotion around peers.

We conclude that despite the universality of the human capacity to feel emotion, the ways in which emotion is displayed are bound by culture-specific values and beliefs. For accurate and complete knowledge of children’s emotional development, early childhood research must reflect the diversity that exists in society and document emotion socialization around the world. More broadly, our understanding of the human condition, including learning, communication, and development, is incomplete without the inclusion of linguistically and culturally diverse groups in scientific research^70^.

## Methods

### Participants

Participants were 21 Thai mother-child dyads in Thailand and 21 American mother-child dyads in the United States. Children were 4-year-old preschoolers (Thai *M*_age_ = 53.19 months, *SD* = 4.42 months; American *M*_age_ = 52.43 months, *SD* = 3.75 months). Mothers’, children’s, and fathers’ background information were obtained using the *Language Experience and Proficiency Questionnaire* (LEAP-Q^71^). Socioeconomic status (as measured by maternal and paternal years of education) of the Thai and American families in our sample did not differ (*p*s > 0.05). Informed consent and child assent were obtained from all participants.

Thai families were recruited in Bangkok, Thailand through the first author’s contacts at preschools in Bangkok and through snowball sampling. American families were recruited in Chicago, United States via Northwestern University participant databases. Families from both cultural groups resided in urban areas. All 21 Thai mother-child dyads were Asian and of Thai ethnicity, which is representative of the homogeneity in Thai population^72^. Of the American mother-child dyads, 19 were White and 2 were African American. To assess participants’ cultural identities, Thai and American mothers answered a question in the LEAP-Q about cultural identification. On a 0 (no identification) to 10 (complete identification) scale, all Thai mothers reported identifying with the Thai culture (range: 7 to 10, mode: 10). All American mothers reported identifying with the American culture (range: 7 to 10, mode: 10). Additionally, we collected self-reported measures of language experience, which have been shown to predict cultural identification^73^. Both Thai and American mothers reported spending almost the entirety of their lives immersed in their respective L1-speaking countries (Thailand/United States), high exposure to their L1 (Thai/English), and high L1 proficiency. See Table 6 for detailed sociodemographic information about the Thai and American participants, including age, education, total number of children, birth order, cultural identification, and linguistic profiles (number of years immersed in L1-speaking country, exposure to L1, and proficiency in L1).

**Table 6:** Demographic background of Thai and American families.

	Thai mean (SE)	American mean (SE)
Age (years)		
Children	4.43 (0.08)	4.37 (0.07)
Mothers	37.66 (0.95)	37.16 (1.20)
Fathers	40.03 (1.12)	39.01 (1.36)
Education (years)		
Mothers	18.55 (0.67)	18.00 (0.77)
Fathers	19.20 (1.33)	17.81 (0.68)
Percent of parents with at least college degree		
Mothers	90.48	90.48
Fathers	80.00	100.00
Percent of target children who were first-borns	47.62	52.38
Total number of children in household	1.57 (0.14)	2.04 (0.17)
Maternal cultural identification		
Identification with L1 culture^a^	8.86 (0.22)	9.55 (0.20)
Years immersed in L1-speaking country	36.97 (1.09)	36.07 (1.24)
Current exposure to L1^b^	91.43 (1.61)	98.81 (0.43)
Self-reported L1 proficiency^c^	9.13 (0.19)	9.46 (0.13)

^a^Cultural identification score was measured on a 0 (no identification) –10 (complete identification) scale.

^b^Exposure was reported in terms of percentage per day.

^c^Proficiency was measured on a 0 (none) –10 (perfect) scale.

### Procedure

Experimental protocols were approved by the institutional review board at Northwestern University and were in accordance with the relevant guidelines and regulations. During a preliminary visit, both mothers and fathers filled out questionnaires regarding their own background. Mothers also reported demographic information about their child. In a subsequent visit, mother-child dyads were videotaped interacting at home. Three tasks were used to obtain language samples from mothers and their children: prompted reminiscing, book sharing, and toy play. Afterwards, the researcher elicited personal narratives from each child individually. Tasks always occurred in this order and were not counterbalanced to prevent the topic or structure of the wordless book from influencing the reminiscing narratives shared by mothers and children, as well as to avoid the possibility of children not wanting to stop playing with the toys. Breaks between tasks were taken as needed. Each session lasted approximately 45 to 60 min, depending on the breaks taken.

#### Prompted reminiscing (i.e., joint reminiscing between mother and child)

The current study used word prompts to elicit mother-child personal narratives. Word prompts have been shown to be effective at eliciting memories^74,75^. Mothers were told that because it might be difficult to come up with many stories on request, they would be given words to facilitate the reminiscing process. The following two sets of 11 word prompts were used: (Set 1) blood, cat, airplane, school, lunch, boat, laughing, blanket, butterfly, holiday, and birthday, (Set 2) doctor, dog, car, yard, dinner, zoo, friend, kitchen, spider, summer, and party. Their Thai translations, respectively, are: (Set 1) เลือด, แมว, เครื่องบิน, โรงเรียน, อาหารเที่ยง, เรือ, การหัวเราะ, ผ้าห่ม, ผีเสื้อ, วันหยุด, and วันเกิด (Set 2) หมอ, หมา, รถ, สนาม, อาหารเย็น, สวนสัตว์, เพื่อน, ครัว, แมงมุม, ฤดูร้อน, and งานเลี้ยง. Mothers received one of the two sets of prompts in the language that they knew.

#### Book sharing

Mothers were asked to share with their children a wordless picture book and were instructed to share the story as they typically would share picture books. Half of the mother-child dyads read *Frog, Where Are You?*^76^, while the other half read *Frog Goes to Dinner*^77^, in their respective language.

#### Toy play

Mother-child dyads were given a toy set consisting of farm animals. Mothers were instructed to play with their children as they normally would and to help their children play with as many toys as they were interested in. The same set of toys were used for both the Thai and American groups.

#### Child personal narrative (i.e., minimally scaffolded narratives with the researcher)

In contrast to the other three tasks, the child personal narrative task was designed to elicit conversation from the children without the linguistic input and support from their mothers. During this activity, the researcher asked children about personally experienced events, prompting questions related to injuries (e.g., “tell me what happened when you got hurt”) and evening routine (“what do you usually do in the evening with your family?”) These questions have been commonly used during the child personal narrative task because they tap into children’s most salient memories^78,79^. The interviewer only provided neutral responses (e.g., “can you tell me more?” or “what else do you remember?”) to minimally scaffold the children^78,79^. The same prompts were used with all children.

### Coding and data analysis

Video recordings were transcribed using Codes for the Human Analysis of Transcripts format^80^. Native speakers of Thai and English transcribed all conversations in their respective languages. Coders were blind to the hypotheses and were trained using the same coding manual. Disagreements were discussed until consensus was reached. Intercoder reliability was established between the coders on 20% of the transcripts using Cohen’s Kappa for all measures: Task 1 κ = 0.94 for Thai coders and κ = 0.93 for English coders; Task 2 κ = 0.88 for Thai coders and κ = 0.93 for English coders; Task 3 κ = 0.96 for Thai coders and κ = 0.95 for English coders; Task 4 κ = 0.90 for Thai coders and κ = 0.93 for English coders.

Three types of emotion measures were coded: (1) emotional intensity, (2) use of emotion words (positive and negative), and (3) display of emotion behaviors (positive and negative). See Table 7 for a list of the emotion measures with their corresponding examples. More detailed descriptions of each emotion measure are outlined below:

1. *Emotional intensity*: a global emotional intensity judgment was made per task, factoring in the emotional content of conversation (the extent to which narratives revolved around mothers’, children’s, book characters’, or toy pieces’ emotions) and the emotional intensity in the voice (how animated or excited the mothers and children were). Emotional intensity rating was on a scale of 1–7 (1 = *no emotion*, 2 = *very low*, 3 = *low*, 4 = *moderate*, 5 = *high*, 6 = *very high*, and 7 = *extremely high emotion intensity*). For the prompted reminiscing, book sharing, and toy play tasks, emotional intensity was rated based on the dyadic unit. For the child personal narrative task, emotional intensity was rated based on the child alone.
2. *Use of emotion words*: based on a previously developed coding scheme^81^, emotion words were defined as words that described a positive or negative feeling, such as *happy* or *sad*. Evaluative words (e.g., interesting) or emotionally laden words (e.g., death) were not coded. Emotion words were coded at the word level and could be coded multiple times per utterance (e.g., “you were *scared* [negative emotion word] because daddy was so *mad* [negative emotion word]”). Emotion words were coded and tallied separately for the mothers and the children. During the child personal narrative task, only emotional words produced by the child were coded.
3. *Display of emotion behaviors*: facial expressions or actions that conveyed positive (e.g., *laughing*, *high fiving*, *hugging*) or negative emotions (e.g., *crying*, *frowning*, *brow furrowing*) were coded. Emotion behaviors were coded and tallied separately for the mothers and children. During the child personal narrative task, emotional behaviors were coded only for the child.

**Table 7:** Emotion measures and corresponding examples.

Emotion measures	Examples
Words	
Positive	You were so *excited* to go to the zoo I *love* butterflies! Grandma was *happy* to see you
Negative	I’m *scared* of blood You *hate* broccolis Daddy was *mad*
Behaviors	
Positive	You are so funny! *laughs* You are my sweet baby *hugs* Great job! *high fives*
Negative	That was not nice *frowns* Stop laughing at me! *cries* I’m tired now *furrows brows*

Analyses of variance (ANOVAs) were run separately for each of the three emotion measures in each of the four tasks. Average emotional intensity rating for the *dyadic unit* during prompted reminiscing, book sharing, and toy play was submitted to 2 (cultural group) × 2 (child gender) ANOVAs to determine if there was a significant difference in emotional intensity as a function of the cultural group or child gender. Average emotional intensity rating during the child personal narrative was calculated for the *children* and submitted to a 2 (cultural group) × 2 (child gender) ANOVA.

The average percentage of emotion words (calculated by dividing the total count of emotion words by total number of words mothers produced and multiplying by 100) was submitted to a 2 (cultural group) × 2 (child gender) ANOVA to determine if there was a significant difference in *maternal* use of emotion words as a function of the culture or child gender. The same analytic approach was used to determine if there was a significant difference in *child* use of emotion words.

The average percentage of emotion behaviors (calculated by dividing the total count of emotion behaviors by total number of words mothers produced and multiplying by 100) was submitted to a 2 (cultural group) × 2 (child gender) ANOVA to determine if there was a significant difference in *maternal* display of emotion behaviors as a function of the culture or child gender. The same analyses were run to determine if there was a significant difference in *child* display of emotion behaviors.

Using the lowest and highest values of effect sizes, post-hoc power analyses revealed that power ranged from 0.60 to 0.84.

### Ethics and inclusion statement

The first author involved in designing, implementing, and disseminating the present work is a Thai native speaker who grew up in Thailand. Relevant research on emotion socialization in Thailand has been reviewed and referenced in the manuscript. The lack of research on parent-child emotion socialization in Thailand demonstrates the importance of the current study to the local community. Because this work did not involve external collaborators, ethics approval was obtained from the authors’ institution.

## Data Availability

The data analyzed in the current study are available from the corresponding author on reasonable request.
